# A Source Area Approach Demonstrates Moderate Predictive Ability but Pronounced Variability of Invasive Species Traits

**DOI:** 10.1371/journal.pone.0155547

**Published:** 2016-05-17

**Authors:** Günther Klonner, Stefan Fischer, Franz Essl, Stefan Dullinger

**Affiliations:** Department of Botany and Biodiversity Research, Faculty of Life Sciences, University of Vienna, Vienna, Austria; University of A Coruña, SPAIN

## Abstract

The search for traits that make alien species invasive has mostly concentrated on comparing successful invaders and different comparison groups with respect to average trait values. By contrast, little attention has been paid to trait variability among invaders. Here, we combine an analysis of trait differences between invasive and non-invasive species with a comparison of multidimensional trait variability within these two species groups. We collected data on biological and distributional traits for 1402 species of the native, non-woody vascular plant flora of Austria. We then compared the subsets of species recorded and not recorded as invasive aliens anywhere in the world, respectively, first, with respect to the sampled traits using univariate and multiple regression models; and, second, with respect to their multidimensional trait diversity by calculating functional richness and dispersion metrics. Attributes related to competitiveness (strategy type, nitrogen indicator value), habitat use (agricultural and ruderal habitats, occurrence under the montane belt), and propagule pressure (frequency) were most closely associated with invasiveness. However, even the best multiple model, including interactions, only explained a moderate fraction of the differences in invasive success. In addition, multidimensional variability in trait space was even larger among invasive than among non-invasive species. This pronounced variability suggests that invasive success has a considerable idiosyncratic component and is probably highly context specific. We conclude that basing risk assessment protocols on species trait profiles will probably face hardly reducible uncertainties.

## Introduction

The search for traits which define successful invaders is one of the fundamental issues in invasion biology [1–3]. Identifying such traits is not only of scientific interest but would also facilitate predictions about which species might cause ecological or socio-economic problems upon introduction and hence help to improve proactive management. Researchers have thus used various approaches to detect such ‘invasion traits’, or trait values, like the comparison of invasive and/or non-invasive alien species with native ones in the introduced ranges, or the comparison of native species that have become invasive elsewhere in the world with those that have not (e.g. [3, 4, 5–9]). For plants, these efforts have shown that attributes like fast growth, maximum size and high dispersal abilities actually tend to be positively related to invasiveness (e.g. [7, 10]). However, the ability of these traits to discriminate invasive from non-invasive species was mostly moderate at best, and an undisputed set of attributes that clearly distinguish invaders has not yet emerged (e.g. [11]).

Inconsistencies among the traits under study, interactions among traits (e.g. [6]) as well as methodological differences (e.g. [10]) might have contributed to the mixed results achieved so far. In addition, the trait profile that makes a species invasive may not necessarily be the same in all contexts (e.g. [12]). As an example, recent studies comparing functional traits among invasive and co-occurring native species have demonstrated alien species are functionally distinct from native communities, i.e. they are functionally more distant from the native community than the native species are among themselves [13, 14, 15, 16]. These findings support the idea that successful invasion into native communities requires some ‘empty niches’ or parts of the trait space not already occupied by the resident species [16–18]. As native communities are diverse in their trait profiles, the attributes and attribute combinations that make species invasive may therefore be expected to vary too, either in different native communities within one region or in different regions that have different species pools.

Interestingly, however, studies searching for ‘invasion traits’ have so far concentrated on comparing mean trait values among successful invaders and different comparison groups but have rarely focused on trait variability within the group of invasive species. Individual studies hence show that the average invasive species differs, or does not differ, from the average species of some comparison group with respect to particular traits (e.g. [3, 10, 19]), but provide little information about the dispersion of trait values within the two groups. Knowledge of this variability is clearly of importance for our understanding of invasions as large variability likely implies complex and highly context-dependent causation while low variability indicates a predominant, generic impact of only few factors and processes on invasive success. Knowing this variability is also essential for managing and controlling plant invasions as risk assessment protocols are often based on traits [20] and the ability to recognize potential invaders from their trait profiles decreases with increasing variability of these traits. In other words, if trait variability among invaders is large, trait-based risk assessment protocols become less reliable because misclassifications of risk species will likely increase.

The objective of this study is to expand the focus of ‘invasive trait’-research by combining a screening for potential invasive traits or trait values with an evaluation of how uniform, or variable, the group of invasive species actually is with respect to these potential ‘invasion traits’ as compared to a non-invasive contrast group. Using the non-woody flora of Austria as a study system, we thereby apply a so-called source area approach [7, 17, 18] which focuses on a regional native species pool and compares species that have become invasive elsewhere with those that have not. As compared to the target area approach, i.e. the comparison of invaders and natives in the formers’ invaded ranges, this strategy reduces potential confounding effects of variable evolutionary predispositions [21–23] and differential reachability of invaded ranges for the members of a regional invader pool [7]. We expect that like in similar studies (e.g. [6, 7, 9, 24, 25]), our screening will demonstrate that invasive and non-invasive species differ with respect to at least a subset of traits. With respect to variability in these traits, by contrast, the lack of explicit studies constrains the formulation of clear hypotheses. However, the idea that potential invaders may be recognizable from their traits suggests that being a successful invader requires a certain, distinct trait profile, whereas no such constraints apply to the set of non-invasive species. Our working hypothesis is hence that trait variability is lower among invasive than among non-invasive species.

## Methods

### Data collection

Our analysis focused on the native, non-woody terrestrial vascular plant flora of Austria because knowledge about traits and habitat affiliation of this source area is consistent and relatively complete. The flora of Austria is rich in species as the country covers all the different biogeographical regions of Central Europe [26]. In addition, European regions are particularly suited for a source area approach as Europe has historically served as a main donor of invasive plants for the rest of the world [8, 27].

From the total set of vascular plants in Austria we restricted our analysis to the subset of terrestrial, non-woody spermatophytes, i.e. we excluded the life forms of phanerophytes, chamaephytes and hydrophytes as well as ferns, clubmosses and horsetails, because concentrating on particular taxonomically or ecologically defined plant groups or growth forms was suggested to be more promising when searching for invasion traits [3, 28]. Further, we removed all species not indigenous to Austria, both neophytes and archeophytes, to adhere to a source area approach in a strict sense. Moreover, we excluded 251 species from taxonomically insufficiently resolved, often apomictic genera like *Alchemilla* or *Hieracium* for which taxonomic treatment is likely to vary among different floras and invasive species lists. Finally, we could not consider species which were not represented in the trait databases used (see below). These successive reduction steps left us with a set of 1402 species.

For these 1402 species we searched for trait information in several different trait databases, namely the Ecological Flora Database [29], BiolFlor [30], LEDA [31], CLO-PLA [32], Fischer et al. [33] and Jäger et al. [34]. Based on considerations of both relevance (e.g. [6–9, 24, 25]) and data availability, we selected eight biological traits and differentiated the following trait states (1) Life form after Raunkiaer [30]: therophyte, geophyte, hemicryptophyte and hemiphanaerophyte. (2) Life span as an indicator of generation time [30]: annual, biennial, perennial-pollakanthic, perennial-hapaxanthic. (3) Mating system as an indicator for uniparental reproduction [30]: allogamous, autogamous, mixed mating. (4) Pollen vector [30, 34]: wind, insects, self-pollination, cleistogamy. (5) Type of reproduction [30] according to [30]: by seed and vegetative, by seed, mostly by seed and rarely vegetative, mostly vegetative and rarely by seed, vegetative. (6) strategy type [30] according to [35]: competitors, competitors/ruderals, competitors/stress-tolerators, competitors/stress-tolerators/ruderals, ruderals, stress-tolerators, stress-tolerators/ruderals. (7) Ellenberg’s N-indicator value as a proxy of the species’ ability to exploit high nutrient values [30, 34]: 1 (nutrient poor) to 9 (nutrient rich). Ellenberg’s N-indicator values represent an ordinal classification of plants according to the position of their realized ecological niche along a gradient of nutrient availability. Although they are based on expert judgement and refer to the species’ niche optimum only, they have been proven reliable and useful ecological indicators in many studies (e.g. [36]). (8) maximum plant height, according to [33]: to homogenize data types, we discretized this only numeric trait into (ordered) factor levels (S1 Table). In addition, we collected data on traits related to abundance in the source area and to use as an ornamental species, both indicators of the likelihood of being transported elsewhere and hence of propagule pressure (e.g. [7]), and to habitat requirements: (9) frequency of species within Austria [33]: very rare, rare, dispersed, frequent, very frequent; (10) ornamental use in Austria [37]; (11) presence in agricultural/ruderal habitats [33]: yes/no; (12) presence around aquatic habitats [33]: yes/no; (13) presence below the montane belt as unintentional transportation indicator [33]: yes/no; (14) number of altitudinal belts as an indicator of climatic tolerance [33]. For statistical analysis these traits were classed into five groups: (A) life history (1–2), (B) reproduction (3–5), (C) competitiveness (6–8), (D) habitat use (11–14) and (E) propagule pressure (9–10). Table 1 gives an overview about which trait was assigned to which trait group. We also recorded the family to which each species belongs to avoid possible evolutionary dependence among species in the statistical analyses [33]. As the response variable, i.e. as an indicator of which species of the Austrian flora are actually invasive somewhere else, we used the classification of a species as an environmental weed in the latest edition of Randall’s Global Compendium of Weeds [38]. Weediness sensu this source implies a certain economic or environmental impact [39], i.e. a state of invasion that is going beyond mere establishment of a species as part of a regional flora, i.e. naturalization, and which is usually associated with considerable frequency/abundance. With reference to the state-classification of alien invasions by Richardson et al. [40], ‘weediness’ is hence close to the latest state named ‘invasion’ (in contrast to ‘introduction’ and ‘naturalization’). In our analysis, we concentrate on this state of invasion because at the time of this study, Randall’s Global Compendium of Weeds [36] was the only comprehensive global list of invasive species. Finally, all species not included in Randall [36] were classified as non-invasive.

**Table 1:** Traits and trait groups used to explain invasiveness.

trait group	trait	effect	R²	AIC
intercept-only model		-	0	1426.3
life history			10.4	1369.7
life form		geophyte	1.1	1425.8
life span		annual	8.6	1383.5
reproduction group			*	1417.8
reproduction		seed veg	*	1417.9
mating system		-	*	1426.5
pollen vector		-	*	1425.1
competitiveness			20.9	1298.9
strategy type		c in general, r, sr	8.7	1375.0
N-value		with increasing rank	14.2	1333.1
maximum plant height		with increasing height	4.8	1398.4
habitat use			34.6	1199.8
occurrence in agricultural or ruderal habitats		yes	13.0	1298.1
occurrence around aquatic habitats		yes	1.9	1411.3
occurrence under the montane belt		yes	29.2	1285.1
number of altitudinal belts		with increasing number	2.7	1404.2
propagule pressure			12.7	1316.8
frequency		very frequent	12.4	1327.3
ornamental use		yes	2.7	1406.1

The table presents the traits tested, their combination to groups as well as the marginal *R²* [42, 43] and Akaike information criterion (AIC) values of models using these traits and trait groups to explain invasiveness of Austrian non-woody vascular plants in other parts of the world. “Effect” indicates which trait levels promote invasiveness most strongly.

* R^2^ cannot be calculated due to convergence problems.

### Statistical analysis

As we did not have a phylogenetic tree of our species available, we used generalized linear mixed effects models (GLMMs) with a logistic link function and family as a grouping variable to explore which of the traits collected were significantly related to the probability of a species becoming invasive. To avoid overfitting the GLMMs by using 14 different traits with partly multiple factor levels, and all their possible interactions as predictors, we performed this search for ‘invasion traits’ in several steps. First, we fitted a univariate model for each of the 14 traits separately. Then, we combined the 14 traits into five groups (Table 1) and fitted multiple GLMMs for each particular group to assess the relative importance of the single traits within these groups when other traits of the same group are simultaneously accounted for (in these models we fitted a random intercept for plant family only, because model parameter search algorithms did not converge for more complex models). Finally, we summarized the variables in each group by the first axis of a multiple correspondence analysis (CA) and then searched for the most parsimonious model using all five CA-axes and all their possible two-way interactions as potential independent variables. We used CA because almost all of our variables contain trait information as categorical rather than numeric values. To make the fixed effect estimates of the axes from the five different CAs directly comparable we standardized all 1^st^-axis-values and tested them on collinearity before running the GLMMs. The most parsimonious model was then selected based on the Akaike information criterion (AIC). Since the AIC only provides an estimate of the relative fit of alternative models [41] we also calculated a marginal *R*^*2*^ [42, 43] which describes the proportion of variance explained by the fixed factors of the GLMMs alone.

We used measures of functional diversity to assess the variability of invasive and non-invasive species in multidimensional trait space. The measurement of functional diversity of communities or species groups has recently undergone important progress and several distance-based functional diversity indices have been developed [44–46]. Among those, we selected two different ones which both measure the dispersion of species in trait space [46]: Functional Richness (FRic) is based on calculating convex hulls in multidimensional trait space, i.e. the minimum geometry that contains all species as an indicator of the volume of functional space occupied by a community [45]. FRic is hence a measure of multidimensional trait variability analogue to the range of values of a single variable. Functional Dispersion (FDis), by contrast, the mean distance of individual species to the centroid of all species in multidimensional trait space, is rather analogous to the variance of individual variables and hence less sensible to outliers than FRic [46]. Here, we calculated both indices by means of the FD-package in R [46]. The FD-package computes diversity indices from semi-quantitative and qualitative variables by replacing them by the axes of a principle coordinate analysis (PCoA) of a Gower dissimilarity matrix. Negative eigenvalues of PCoA-axes were handled by adding the minimum constant to the distances that makes all eigenvalues positive [47].

To assess if multidimensional trait variability among invasive alien species is smaller, equal to, or even higher than among non-invasive species we compared the FRic and FDis of the subset of species classified as invasive against those of a re-sample (without replacement) of an equal number of species from the total pool of all 1402 species. We repeated re-sampling 1000 times and evaluated if the empirical values of the invasive group are within the 0.95 confidence interval of the 1000 values of these resamples. We conducted this comparison with FRic and FDis calculated for the overall set of traits as well as for the subsets of traits found to distinguish invasive and non-invasive species in the previous analyses [48].

All statistical analyses were conducted in R 3.1.1 [49] using contributed packages lme4 1.1–6 [50], ade4 1.6–2 [51], piecewiseSEM 1.0.0 [52] and FD 1.0–11 [53].

## Results

From the set of 1402 species 305 were classified as invasive elsewhere whereas 1097 were not considered to be invasive aliens anywhere in the world (S3 Table).

### Which traits are most related to invasiveness?

The traits analysed showed different effects on the species’ probability of becoming invasive outside their native range (Table 1 and S1 Fig). Traits indicating the use of particular habitats were most useful for discriminating invasives, in particular “occurrence below the montane belt” and “occurrence in agricultural or ruderal habitats”. Among the other variables tested preference of sites with high nitrogen availability, frequency of the species within Austria, and a competitive or ruderal strategy correlated with invasiveness most clearly. However, none of the single trait models was convincingly distinguishing native species, which are either invasive or non-invasive elsewhere. Summarizing traits into groups and calculating multiple models demonstrated that the trait group ‘reproduction’ explained invasiveness worst (highest AIC value), while the trait groups ‘habitat use’ and ‘competitiveness’ had highest predictive abilities (Table 1).

Among models which used CA axes (corresponding to the different trait groups) the best one not including interactions explained 40.35% of the variance (see AIC-values in S2 Table). All groups except ‘reproduction’ were included in this model with ‘habitat use’ having by far the strongest and ‘life history’ the weakest effects. Including interactions further improved models slightly. The best model had a marginal *R²* = 45.93%, but reduced the AIC by only ΔAIC = 4.2 as compared to the best model without interactions; it also included only one of all possible interactions, namely the one among competitiveness and habitat use (Table 2 and S2 Table).

**Table 2:** Best GLMM to explain invasiveness of Austrian non-woody vascular plants in other parts of the world.

	estimate	std. error	z-value	p-value	
AIC = 1128.8; R^2^ = 45.92					
life history	0.28	0.08	3.63	2.85 × 10^−4^	***
competitiveness	0.46	0.09	-4.93	8.21 × 10^−7^	***
habitat use	1.31	0.13	-9.88	2.00 × 10^−16^	***
propagule pressure	0.51	0.08	6.49	8.52 × 10^−11^	***
competitiveness:habitat use	0.24	0.10	-2.51	1.21 × 10^−2^	*

Traits represent first axes of correspondence analyses of the respective trait groups (Table 1) which were standardized before running the GLMMs. Best models were selected based on the Akaike information criterion (AIC) from all possible candidate models (S2 Table). The model’s corresponding marginal R^2^ value [42, 43] and Akaike Information Criterion are also shown. *,*** give information on the p-values significance.

### Variation in trait space

**Fig 1:**
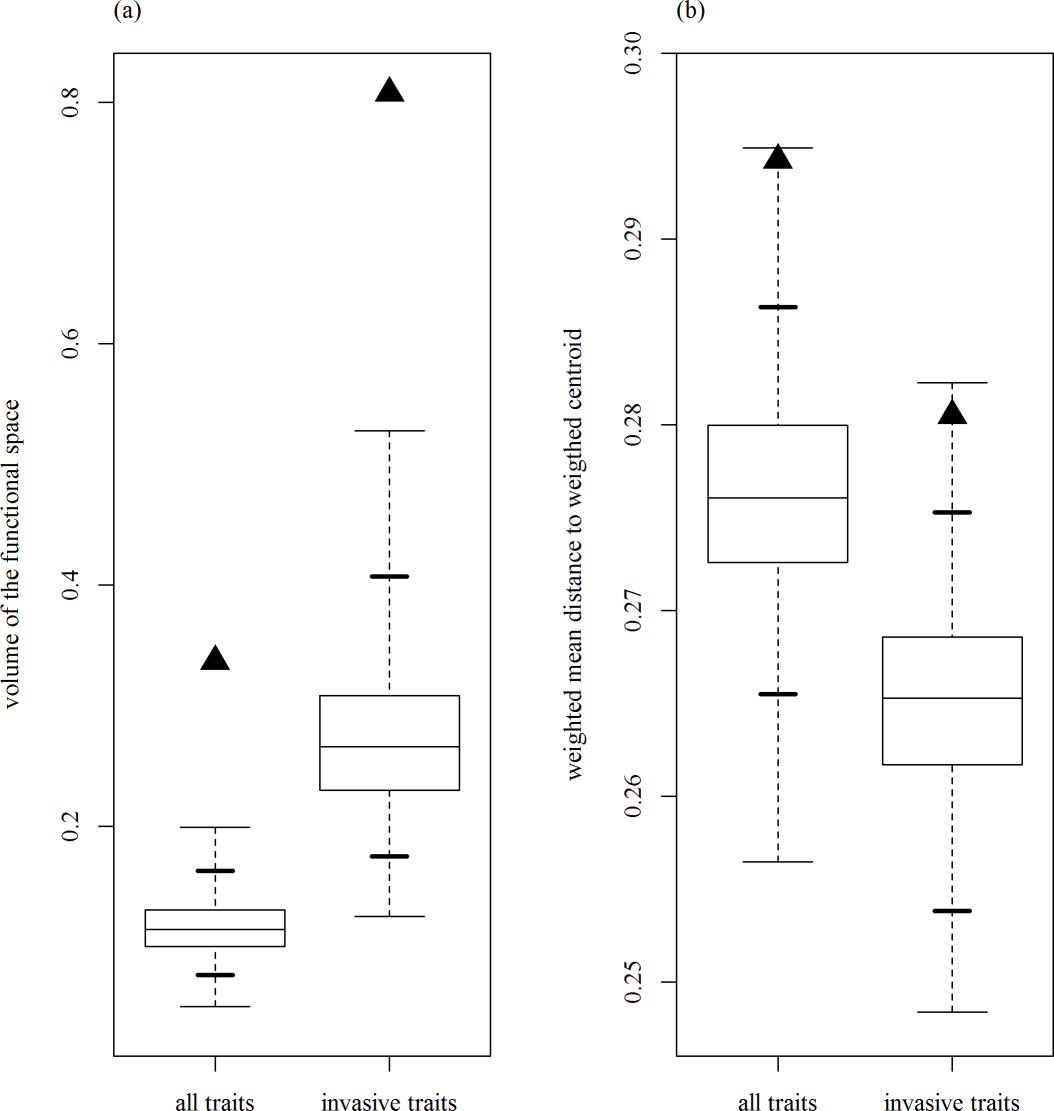
Comparison of functional diversity indices among Austrian plants that are either invasive or not invasive elsewhere in the world. Black triangles symbolize the diversity index values calculated for the group of the 305 invasive plants. The boxplots represent the range of index values calculated for 1000 equally large re-samples from the whole pool of 1402 native species with the bold lines indicating the 0.95 confidence interval of these re-sample based values. Panel (a) represents results for Functional Richness (defined by the volume of the functional space) and panel (b) results for Functional Dispersion (defined by the mean distance in multidimensional trait space of individual species to the centroid of all species), respectively. Label ‘all traits’ give results calculated with the total set of collected traits, label ‘invasive traits’ calculations based on traits that proved useful to distinguish invasive and non-invasive species in the preceding analyses.

According to the previous analyses the traits ‘life form’, ‘mating system’ and ‘pollen vector’ did not improve a random effects model (= an intercept-only model) by a ΔAIC > 2 and were hence considered not useful to distinguish invasive and non-invasive species [48]. The trait ‘ornamental use’ was not included in this analysis since we did not expect any relationship with functional diversity. When measured by FRic the variation in multidimensional trait space among the species classified as invasive was in general even larger than the variation of a random sample of native plants from the Austrian source pool (Fig 1). This result held independent of whether all traits or only those useful to distinguish invasives were used for calculating FRic (Fig 1A). Using FDis, which is less sensitive to outliers, yields similar results: the functional dispersion was larger for invasive species when calculated either on the whole set of traits or on the set of ‘invasive traits’ (Fig 1B).

## Discussion

In summary, our results demonstrate that, as expected, most of the analysed traits are actually related to their probability of becoming invasive outside their native range to a certain extent. Nevertheless, neither a single trait group nor their combination to one best model allowed for distinguishing invasive aliens with high reliability. In addition, and against our ‘working hypothesis’, we found that multidimensional variability in trait space is even more pronounced among invasive as among non-invasive plants.

### Single trait effects

Our single trait analyses suggest that attributes of the species related to the use of specific habitats are among those most closely correlated to invasion success. In particular, plants which occur in agricultural and ruderal habitats are more likely to become invasive outside their native ranges. This correlation is probably driven by a combination of different factors with both pre-adaptation to sites particularly sensitive to invasions and propagule pressure playing key roles. On the one hand, agricultural and ruderal habitats are not only donor but also important recipient habitats of invaders (e.g. [8, 54, 55]) because, first, they are characterized by a high frequency of human disturbance events which keep competitive resistance against invasion low; and, second, because the surroundings of sites of introduction, and hence of high propagule pressure, are usually characterized by high human population density and intense land use, i.e. by a high incidence of ruderal and agricultural habitats (e.g. [56]). As a corollary, biological traits that are selected by the conditions prevailing in these habitats, such as short life span, efficient reproduction by seeds and, in general, a ruderal life history strategy [35] are also among those positively associated with invasion success in our as well as in other studies (e.g. [7, 17, 57]). On the other hand, the preference of agricultural and ruderal habitats in the native range might also be related to invasions because of a sampling effect–the higher human population and the more intense usage also increase the likelihood of exportation of propagules from these areas. This sampling effect, and hence propagule pressure, is clearly also driving the influence of species frequency on invasiveness (cf. [7]). By contrast, the fact that invasive species mainly grow in lowlands of their Austrian range might again have to do with both matching between native and invaded habitats and the concentration of introduction foci in lowland areas. Alexander et al. [56] have, for example, suggested that the bias of introduction events towards lowland areas might prevent first establishment of mountain plants in the introduced range and hence also their expansion into more suitable sites at higher elevations–and thus explain why mountains are less affected by invasions. Moreover, propagules of mountain plants probably have a lower uptake probability, at least in temperate regions [8, 27], i.e. they are more rarely transported by humans over long distances because mountains are more sparsely populated and less intensively used than lowland areas. Thus, mountain species are probably not only less likely to establish at (typical) introduction sites, they are also less likely to reach them.

Regarding biological traits we found those associated with high competitive ability, like maximum height and ability to exploit high nutrient supply levels to show strongest correlation with invasiveness. These results are in line with findings of experimental studies which showed invasive species to usually have high growth rates, tall size as well as high leaf and shoot allocation [3, 58–60]. Similar to Pyšek et al. [7] our data suggest that in this context invasiveness is less conferred by a particular trait like the often studied plant height (e.g. [19, 61, 62])–which has a significant, but relatively low effect on Austrian species becoming invasive elsewhere–but rather by a life history syndrome composed of a whole set of traits [6]. The ability to translate high nutrient input into fast growth is an important component of this trait combination which distinguishes C from S-strategists in particular [35]. The clear effect of N-indicator values on invasiveness hence fits well with the one of the competitive strategy type and is in line with other studies demonstrating that invasive species usually prefer sites with high nitrogen supply rates [24, 59, 63]. In addition, plants introduced into new regions often shift towards faster growth strategies because of a reduction in the top-down constraint imposed by herbivores [64], and thus may effectively use high nitrogen supplies. However, high nutrient availability is also characteristic for ruderal and agricultural habitats and the relative roles of preadaptation, or habitat matching, and propagule pressure in driving the N-effect on invasiveness are hence again hard to disentangle.

For some of the traits tested expected relationships with invasiveness were actually not detected at all. For example, autogamous species should have an advantage during invasions because they do not depend on mating partners for establishment and spread [65, 66], but we did not find any significant effect of mating systems on the invasiveness of Austrian plants. We hypothesize that the lack of such an effect is partly due to our focus on invasiveness, i.e. on the latest stage of the invasion process, while the advantage of self-compatibility may particularly be relevant during early stages when populations of the invading species are still small and Allee-effects might play a prominent role [67]. Additionally, species may also change traits from native to invasive ranges. In some species, invasions have for example been associated with shifting investment from sexual to vegetative reproduction, or vice versa (e.g. [68]). Such shifts potentially mask the relationship between traits and invasive success and must go undetected with a source area approach. This possible shortcoming underlines that reaching unambigous conclusions in invasion biology will often require the combination of different approaches [3].

### Multiple trait models

Combining traits into groups and building a multiple model that integrated all these groups improved the distinction between invasive and non-invasive plants considerably. The best model, including interactions, explained ~ 46% of the variance. This value puts our model closer to the one reported by Pyšek et al. ([7]: 43% of variance explained by their best model) than to those reported by Küster et al. ([6]: c. 25% of variance explained by their best model). Both studies analyzed the relative importance of different trait sets for species’ invasion success but either used a source area [6] or a target area approach [7]. Further Pysek et al. [7] address the stage-structure of the invasion process while Küster et al. [6] highlight the importance of incorporating trait interactions when testing for traits that promote invasion. The lower success in the latter study might result from the fact that Küster et al. [6] used a target area instead of a source area approach and that they focused on all naturalized, and not only on invasive species. Actually, the effect of biological traits on naturalization rather than invasiveness has been found to be considerably lower by Pyšek et al. [7], too. Moreover, in agreement to Küster et al. [6, 69], interactions among traits had an effect on invasiveness in our models, although this effect was rather weak. We speculate that these interactions might be even more relevant when focusing on a specific region of introduction, like the target area approach does (e.g. [6]), than when pooling invasiveness across all the different adventive ranges of a large set of species like in the source area approach. We assume this to be the case because, with the latter focus, interactions of traits with highly variable abiotic and biotic conditions in the introduced ranges may become much more important than interactions among the traits themselves (cf. [24, 25] and discussion below).

### Multidimensional trait variability

Following the idea that invasive species may be identifiable from distinct trait profiles, we had expected that trait variability among invasive species is smaller than among non-invasives. Our results did not corroborate this expectation. By contrast, invasive species appeared even more variable in their trait profiles than the non-invasive contrast group. One explanation of this result may be that important determinants of invasive success, which interact with the evaluated traits, are missing from the model. Among these determinants, quantitative data on introduction efforts or propagule pressure and hence establishment opportunities, might be particularly important [70]. In addition, high variability in ‘invasive traits’ is also consistent with the idea that traits conferring invasive success depend on specific ecological settings in the recipient area. With respect to different habitat types such specificity of ‘invasion traits’ has already been discussed (e.g. [3, 24]). As an example, successful invaders might rather be ruderal strategists sensu Grime [35] when spreading into highly disturbed agricultural or urban habitats while a competitive strategy might be more promising when disturbance frequency is lower, like in many semi-natural or natural habitats. Even an S-strategy might be helpful in particular cases, e.g. when species are invading regions characterized by cold temperatures or low water availability. Recent studies have moreover shown that there is a strong negative correlation among the trait profiles of invaded communities and the attributes necessary to invade these communities at local scales [13, 14], while at larger spatial scales this correlation may switch into a positive one [16]. These results strongly suggest that, for becoming a successful invader, differences in trait profiles from those prevailing in the native communities might be more important than specific trait values *per se*. In a source area approach, species invasive all over the world are simultaneously considered. The variation among recipient areas and invaded communities is hence large. In light of these recent studies which emphasize the strong context dependence of invasive success the pronounced trait variability that we found among invaders in our source areas approach appears hence less surprising.

From the perspective of invasive species management and risk assessment, our results imply that attempts to recognize potential invaders based on traits will remain challenging because high variability is likely associated with considerable ‘error rates’. If this variability is, as hypothesized, at least partly due to (dis)similarities of successful invaders with native communities an appropriately differentiated approach might actually be promising. In other words, our results question the existence of a distinct trait profile that makes species invasive independent of the abiotic and biotic context. Further they suggest adapting critical trait profiles in risk assessment protocols to the particular environmental conditions and trait profiles of resident native communities as far as possible.

## Supporting Information

S1 Fig Proportions of plants that are invasive/ not invasive in other parts of the world. (PDF)

S1 Table Categorization of ‘maximum plant height’. (PDF)

S2 Table Evaluation of candidate generalized linear mixed effect models based on the Akaike information criterion. (PDF)

S3 Table List of study species analysed and their status of being invasive somewhere outside their native distribution. (PDF)
