# Correction: A Source Area Approach Demonstrates Moderate Predictive Ability but Pronounced Variability of Invasive Species Traits

**DOI:** 10.1371/journal.pone.0172656

**Published:** 2017-02-16

**Authors:** Günther Klonner, Stefan Fischer, Franz Essl, Stefan Dullinger

In the Funding section, the grant number from the funder FWF (Austrian Science Fund) is listed incorrectly. The correct grant number is: I 1443-B25.
