# Supplementary material for: A Source Area Approach Demonstrates Moderate Predictive Ability but Pronounced Variability of Invasive Species Traits
Source: PLoS One. 2016 May 17;11(5):e0155547. doi: 10.1371/journal.pone.0155547 (PMC4871327; doi:10.1371/journal.pone.0155547)
Supplement: S1 Fig — (PDF) [file pone.0155547.s001.pdf]

S1 Fig. Proportions of non-woody vascular plants of the Austrian flora that are invasive (“yes”) or not invasive (“no”) in other parts of the world in the different classes (= levels) of the analysed traits. Given are all 14 used traits in their corresponding trait group: (a) life history, (b) reproduction, (c) competitiveness, (d) habitat use, (e) propagule pressure.

(a)

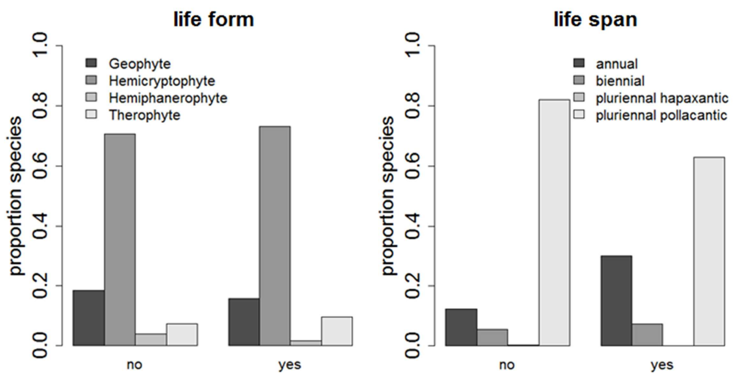

(b)

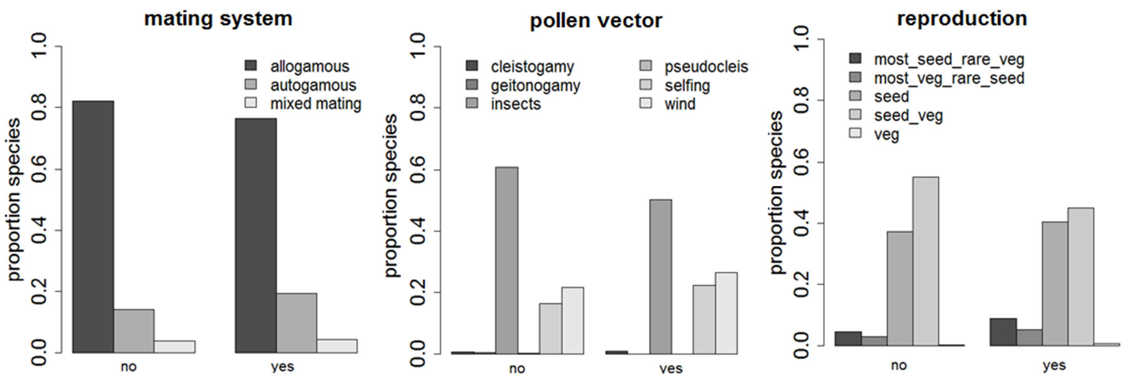

(c)

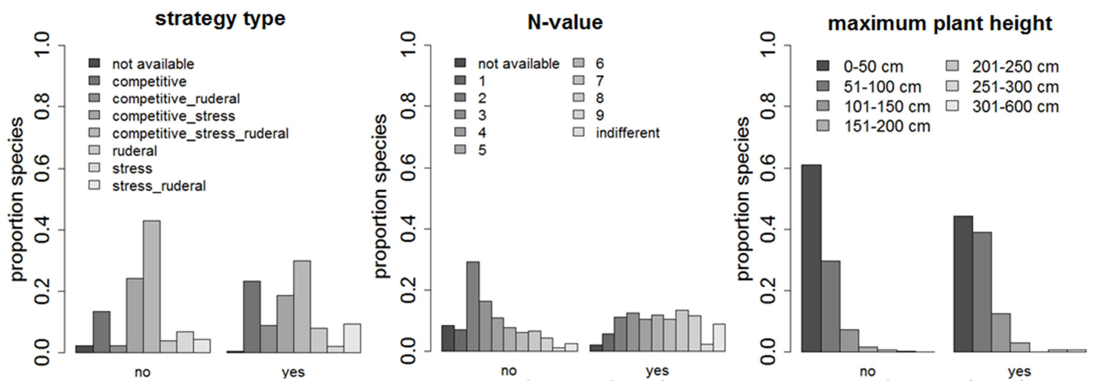

(d)

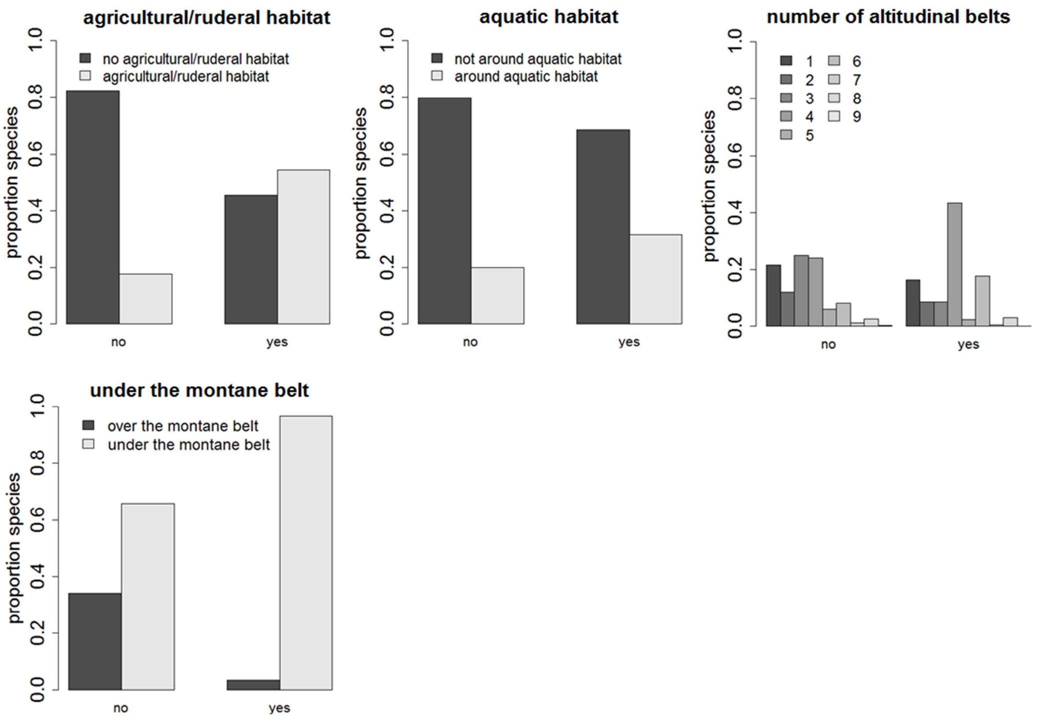
agricultural/ruderal habitat
no agricultural/ruderal habitat
agricultural/ruderal habitat
proportion species
0.0
0.2
0.4
0.6
0.8
1.0
no
yes
aquatic habitat
not around aquatic habitat
around aquatic habitat
proportion species
no
yes
number of altitudinal belts
1
2
3
4
5
6
7
8
9
proportion species
no
yes
under the montane belt
over the montane belt
under the montane belt
proportion species
no
yes

(e)

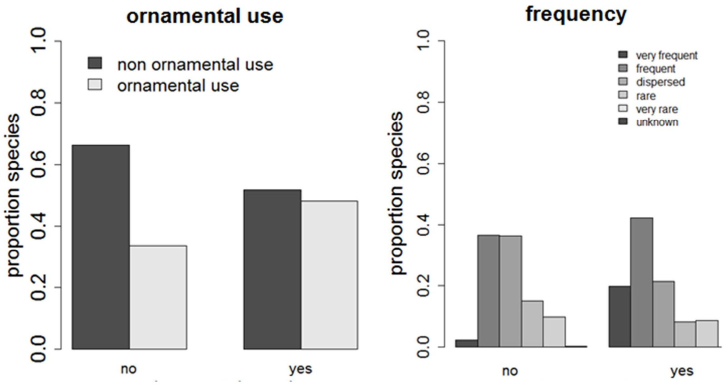
ornamental use
non ornamental use
ornamental use
proportion species
0.0
0.2
0.4
0.6
0.8
1.0
no
yes
frequency
very frequent
frequent
dispersed
rare
very rare
unknown
proportion species
no
yes
