# Supplementary material for: A Source Area Approach Demonstrates Moderate Predictive Ability but Pronounced Variability of Invasive Species Traits
Source: PLoS One. 2016 May 17;11(5):e0155547. doi: 10.1371/journal.pone.0155547 (PMC4871327; doi:10.1371/journal.pone.0155547)
Supplement: S1 Table — (PDF) [file pone.0155547.s002.pdf]

S1 Table. Categorization of 'maximum plant height'.

factor classes	metric values (cm)
1	0-10
2	11-20
3	21-30
4	31-40
5	41-50
6	51-60
7	61-70
8	71-80
9	81-100
10	101-150
11	151-200
12	201-600
