# Supplementary material for: A Source Area Approach Demonstrates Moderate Predictive Ability but Pronounced Variability of Invasive Species Traits
Source: PLoS One. 2016 May 17;11(5):e0155547. doi: 10.1371/journal.pone.0155547 (PMC4871327; doi:10.1371/journal.pone.0155547)
Supplement: S2 Table — (PDF) [file pone.0155547.s003.pdf]

S2 Table. Candidate generalized linear mixed effects models (GLMM) evaluated based on the Akaike information criterion (AIC). Numbers represent variable groups: (1) life history, (2) reproduction, (3) competitiveness, (4) habitat use, (5) propagule pressure. Variables within these groups were summarized by a multiple correspondence analysis and the first axis-values of these were used as predictors in the GLMMs. Bold markings refer to the most parsimonious model both without and with interactions. Also given is a marginal R^2 [42, 43].

**model**	R^2	**AIC**	**model**	R^2	**AIC**
**no interactions**			**no interactions**		
1 + 2 + 3 + 4 + 5	40.28	1134.20	2 + 3	7.30	1371.85
1 + 2 + 3 + 4	37.41	1178.69	2	0.51	1424.17
1 + 2 + 3	9.56	1356.25	2 + 4 + 5	37.65	1156.00
1 + 2	2.27	1411.19	2 + 4	35.47	1200.64
1	2.26	1409.23	2 + 5	10.60	1340.41
**1 + 3 + 4 + 5**	**40.35**	**1133.00**	3 + 4 + 5	39.28	1145.60
1 + 3 + 4	37.44	1176.95	3 + 4	37.20	1180.22
1 + 4	35.48	1198.88	3 + 5	13.11	1322.49
1 + 4 + 5	38.21	1148.46	3	6.04	1379.76
1 + 5	14.58	1312.03	4 + 5	37.61	1157.68
2 + 3 + 4 + 5	39.46	1141.73	4	35.52	1199.15
2 + 3 + 4	37.20	1180.08	5	9.17	1350.47
**model**			**model**		
**with interactions**			**with interactions**		
1*2+3+4+5	40.44	1135.99	2*3+4+5	40.12	1140.38
1*3+2+4+5	40.60	1135.39	2*3+4	37.73	1179.34
1*4+2+3+5	42.37	1131.71	2*3+1+5	19.36	1280.02
1*5+2+3+4	39.92	1134.96	2*3+5	15.48	1307.87
2*3+1+4+5	40.67	1133.33	2*3	7.55	1373.14
2*4+1+3+5	40.62	1135.53	2*4+1+3	37.44	1180.66
2*5+1+3+4	40.47	1135.89	2*4+1	35.50	1202.88
3*4+1+2+5	45.83	1129.91	2*4+3+5	39.65	1143.51
3*5+1+2+4	40.07	1135.15	2*4+3	37.21	1182.08
4*5+1+2+3	41.39	1135.78	2*4+1+5	38.49	1151.63
1*2+3*4+5	45.90	1131.71	2*4+5	37.88	1157.82
1*2+3*5+4	40.14	1136.92	2*4	35.46	1202.64
1*2+4*5+3	41.41	1137.56	2*5+1+3	19.15	1280.88
1*3+2*4+5	40.83	1136.78	2*5+1	14.63	1315.06
1*3+2*5+4	40.65	1137.14	2*5+3+4	39.68	1143.32
1*3+4*5+2	41.51	1137.00	2*5+3	15.26	1308.75
1*4+2*3+5	42.80	1131.22	2*5+1+4	38.28	1152.02
1*4+2*5+3	42.22	1133.60	2*5+4	37.80	1157.76
1*4+3*5+2	42.10	1132.79	2*5	10.63	1342.39
1*5+2*3+4	40.33	1133.99	3*4+1+2	44.44	1172.82
1*5+2*4+3	40.20	1136.00	3*4+1	44.49	1171.11
1*5+3*4+2	45.45	1130.75	3*4+2+5	45.84	1136.21
2*3+4*5+1	41.98	1134.87	3*4+2	44.62	1173.52
2*4+3*5+1	40.31	1136.60	**3*4+1+5**	**45.92**	**1128.75**
2*5+3*4+1	46.12	1131.48	3*4+5	46.08	1139.76
1*2+3+4	37.58	1179.83	3*4	44.80	1173.51
1*2+3	9.64	1357.08	3*5+1+2	19.12	1280.88
1*2+4+5	38.20	1152.06	3*5+1	18.89	1281.51
1*2+4	35.62	1202.17	3*5+2+4	39.29	1141.90
1*2+3+5	19.16	1280.51	3*5+2	14.98	1308.63
1*2+5	14.66	1314.72	3*5+1+4	40.12	1133.93
1*2	2.38	1411.84	3*5+4	39.06	1145.36
1*3+2+4	37.59	1180.28	3*5	12.77	1324.12
1*3+2	9.75	1357.78	4*5+1+2	39.59	1151.63
1*3+4+5	40.70	1134.16	4*5+1	39.59	1149.94
1*3+4	37.63	1178.53	4*5+2+3	41.04	1143.01
1*3+2+5	19.39	1280.22	4*5+2	39.47	1157.15
1*3+5	19.19	1280.86	4*5+1+3	41.39	1134.64
1*3	9.58	1357.13	4*5+3	40.81	1146.96
1*4+2+3	39.40	1177.11	4*5	39.38	1158.90
1*4+2	37.75	1199.03	1*2+3*4	44.65	1173.94
1*4+3+5	42.40	1130.79	1*2+3*5	19.14	1282.51

1*4+3	39.40	1175.50	1*2+4*5	39.64	1153.50
1*4+2+5	40.59	1147.50	1*3+2*4	37.62	1182.26
1*4+5	40.61	1145.96	1*3+2*5	19.38	1282.22
1*4	37.76	1197.05	1*3+4*5	41.77	1135.81
1*5+2+3	18.85	1278.84	1*4+2*3	39.71	1177.03
1*5+2	14.32	1312.16	1*4+2*5	40.62	1149.48
1*5+3+4	39.94	1133.51	1*4+3*5	42.15	1131.82
1*5+3	18.64	1278.98	1*5+2*3	19.04	1279.77
1*5+2+4	37.78	1150.19	1*5+2*4	38.14	1151.30
1*5+4	37.80	1148.32	1*5+3*4	45.52	1129.32
1*5	14.25	1310.81	2*3+4*5	41.68	1141.61
2*3+1+4	37.86	1178.19	2*4+3*5	39.39	1143.75
2*3+1	9.77	1357.55	2*5+3*4	46.17	1137.66
