# Supplementary material for: A Source Area Approach Demonstrates Moderate Predictive Ability but Pronounced Variability of Invasive Species Traits
Source: PLoS One. 2016 May 17;11(5):e0155547. doi: 10.1371/journal.pone.0155547 (PMC4871327; doi:10.1371/journal.pone.0155547)
Supplement: S3 Table — (PDF) [file pone.0155547.s004.pdf]

S3 Table. List of study species analysed and their status of being invasive somewhere outside their native distribution. The species are first ordered according to their status of being invasive outside of their native range, after a classification of species as environmental weeds in the latest edition of Randall's Global Compendium of Weeds [38]. Further species are listed alphabetically.

Species name | env.weed |					
Achillea millefolium	1	Cardaminopsis arenosa	0	Lychnis viscaria	0
Achillea nobilis	1	Cardaminopsis halleri	0	Lycopus europaeus	0
Achillea ptarmica	1	Cardaminopsis petraea	0	Lycopus exaltatus	0
Achnatherum calamagrostis	1	Carduus defloratus	0	Maianthemum bifolium	0
Acinos arvensis	1	Carduus personata	0	Malaxis monophyllos	0
Aconitum napellus	1	Carex acuta	0	Melampyrum arvense	0
Adoxa moschatellina	1	Carex acutiformis	0	Melampyrum cristatum	0
Aegopodium podagraria	1	Carex alba	0	Melampyrum nemorosum	0
Agrostis canina	1	Carex appropinquata	0	Melampyrum pratense	0
Agrostis capillaris	1	Carex atrata	0	Melampyrum sylvaticum	0
Agrostis gigantea	1	Carex baldensis	0	Melica ciliata	0
Agrostis stolonifera	1	Carex bigelowii	0	Melica picta	0
Aira caryophyllea	1	Carex bohemica	0	Melica transsilvanica	0
Ajuga genevensis	1	Carex brachystachys	0	Melica uniflora	0
Ajuga reptans	1	Carex brunnescens	0	Melilotus altissimus	0
Allium carinatum	1	Carex buekii	0	Melilotus dentatus	0
Allium oleraceum	1	Carex buxbaumii	0	Melittis melissophyllum	0
Allium scorodoprasum	1	Carex canescens	0	Mentha dumetorum	0
Allium vineale	1	Carex capillaris	0	Mentha longifolia	0
Alopecurus aequalis	1	Carex caryophyllea	0	Meum athamanticum	0
Alopecurus geniculatus	1	Carex cespitosa	0	Milium effusum	0
Alopecurus pratensis	1	Carex chordorrhiza	0	Minuartia hybrida	0
Alyssum alyssoides	1	Carex curvata	0	Minuartia rubra	0
Angelica sylvestris	1	Carex davalliana	0	Minuartia viscosa	0
Anthoxanthum odoratum	1	Carex demissa	0	Moehringia ciliata	0
Anthriscus sylvestris	1	Carex diandra	0	Moehringia muscosa	0
Apium graveolens	1	Carex digitata	0	Moehringia trinervia	0
Apium nodiflorum	1	Carex dioica	0	Molinia arundinacea	0
Aquilegia vulgaris	1	Carex distans	0	Monotropa hypophegea	0
Arctium minus	1	Carex echinata	0	Monotropa hypopitys	0
Arenaria leptoclados	1	Carex elata	0	Montia fontana	0
Arenaria serpyllifolia	1	Carex elongata	0	Muscari tenuiflorum	0
Arrhenatherum elatius	1	Carex ericetorum	0	Myosotis alpestris	0
Artemisia vulgaris	1	Carex ferruginea	0	Myosotis decumbens	0
Aruncus dioicus	1	Carex firma	0	Myosotis nemorosa	0
Atriplex prostrata	1	Carex frigida	0	Myosotis rehsteineri	0
Barbarea vulgaris	1	Carex fuliginosa	0	Myosotis sparsiflora	0
Bidens cernua	1	Carex halleriana	0	Narcissus radiiflorus	0
Bidens radiata	1	Carex hartmanii	0	Neottia nidus-avis	0
Bidens tripartita	1	Carex heleonastes	0	Nigritella nigra	0
Bothriochloa ischaemum	1	Carex hirta	0	Nigritella rhellicani	0
Brachypodium pinnatum	1	Carex hordeistichos	0	Nigritella rubra	0
Brachypodium sylvaticum	1	Carex hostiana	0	Nigritella widderi	0
Bromus erectus	1	Carex humilis	0	Nonea pulla	0
Bromus hordeaceus	1	Carex limosa	0	Odontites luteus	0
Bromus inermis	1	Carex melanostachya	0	Odontites vernus	0
Bromus racemosus	1	Carex michelii	0	Odontites vulgaris	0
Bryonia dioica	1	Carex microglochin	0	Omphalodes scorpioides	0
Calamagrostis epigejos	1	Carex montana	0	Onobrychis arenaria	0
Calystegia sepium	1	Carex mucronata	0	Onobrychis montana	0
Campanula rapunculoides	1	Carex muricata	0	Onosma arenaria	0
Capsella bursa-pastoris	1	Carex nigra	0	Ophrys apifera	0
Cardamine flexuosa	1	Carex oenensis	0	Ophrys araneola	0
Cardamine hirsuta	1	Carex ornithopoda	0	Ophrys holoserica	0
Cardamine impatiens	1	Carex ornithopodioides	0	Ophrys insectifera	0

Cardamine pratensis	1	Carex otrubae	0	Ophrys sphegodes	0
Carduus crispus	1	Carex pairae	0	Orchis mascula	0
Carex brizoides	1	Carex pallescens	0	Orchis militaris	0
Carex disticha	1	Carex parviflora	0	Orchis pallens	0
Carex divulsa	1	Carex pauciflora	0	Orchis palustris	0
Carex flacca	1	Carex paupercula	0	Orchis purpurea	0
Carex flava	1	Carex pendula	0	Orchis simia	0
Carex lepidocarpa	1	Carex pilosa	0	Orchis spitzelii	0
Carex ovalis	1	Carex praecox	0	Orchis tridentata	0
Carex panicea	1	Carex pulicaris	0	Orchis ustulata	0
Carex paniculata	1	Carex randalpina	0	Oreochloa disticha	0
Carex pilulifera	1	Carex remota	0	Ornithogalum pyrenaicum	0
Carex punctata	1	Carex rostrata	0	Oxalis acetosella	0
Carlina vulgaris	1	Carex rupestris	0	Oxyria digyna	0
Carpesium cernuum	1	Carex secalina	0	Oxytropis jacquinii	0
Carum carvi	1	Carex sempervirens	0	Oxytropis pilosa	0
Catabrosa aquatica	1	Carex spicata	0	Papaver alpinum	0
Centaurea jacea	1	Carex strigosa	0	Paris quadrifolia	0
Centaurea montana	1	Carex supina	0	Parnassia palustris	0
Centaurea stoebe	1	Carex sylvatica	0	Pedicularis elongata	0
Centaurium erythraea	1	Carex tomentosa	0	Pedicularis foliosa	0
Centaurium pulchellum	1	Carex umbrosa	0	Pedicularis oederi	0
Cerastium diffusum	1	Carex vaginata	0	Pedicularis palustris	0
Cerastium dubium	1	Carex viridula	0	Pedicularis recutita	0
Cerastium semidecandrum	1	Carex vulpina	0	Pedicularis rostratocapitata	0
Chelidonium majus	1	Carlina acaulis	0	Pedicularis rostratospicata	0
Chenopodium polyspermum	1	Carlina biebersteinii	0	Pedicularis sceptrum-carolinum	0
Cirsium arvense	1	Centaurea phrygia	0	Pedicularis sylvatica	0
Cirsium oleraceum	1	Centaurea pseudophrygia	0	Pedicularis verticillata	0
Cirsium palustre	1	Centaurea scabiosa	0	Peplis portula	0
Cirsium vulgare	1	Centaurea stenolepis	0	Persicaria amphibia	0
Clinopodium vulgare	1	Centaurea triumfettii	0	Persicaria brittingeri	0
Convallaria majalis	1	Centaurium littorale	0	Persicaria dubia	0
Convolvulus arvensis	1	Centunculus minimus	0	Persicaria hydropiper	0
Corynephorus canescens	1	Cephalanthera damasonium	0	Persicaria minor	0
Crepis tectorum	1	Cephalanthera longifolia	0	Petasites albus	0
Cuscuta epithymum	1	Cephalanthera rubra	0	Petasites hybridus	0
Cynoglossum officinale	1	Cerastium glutinosum	0	Petasites paradoxus	0
Cynosurus cristatus	1	Cerastium pumilum	0	Peucedanum alsaticum	0
Cyperus longus	1	Ceratocapnos claviculata	0	Peucedanum carvifolia	0
Dactylis glomerata	1	Cerinthe glabra	0	Peucedanum cervaria	0
Danthonia decumbens	1	Chaerophyllum aromaticum	0	Peucedanum officinale	0
Daucus carota	1	Chaerophyllum aureum	0	Peucedanum oreoselinum	0
Deschampsia flexuosa	1	Chaerophyllum bulbosum	0	Peucedanum ostruthium	0
Dianthus armeria	1	Chaerophyllum hirsutum	0	Peucedanum palustre	0
Elatine triandra	1	Chaerophyllum temulum	0	Phleum alpinum	0
Eleocharis palustris	1	Chamorchis alpina	0	Phleum bertolonii	0
Elytrigia intermedia	1	Chenopodium bonus-henricus	0	Phleum hirsutum	0
Elytrigia repens	1	Chenopodium botryodes	0	Phleum paniculatum	0
Epilobium angustifolium	1	Chenopodium rubrum	0	Phleum phleoides	0
Epilobium hirsutum	1	Chimaphila umbellata	0	Phleum rhaeticum	0
Epipactis helleborine	1	Chrysosplenium alternifolium	0	Phyteuma betonicifolium	0
Erica tetralix	1	Cicerbita alpina	0	Phyteuma hemisphaericum	0
Erodium cicutarium	1	Cicuta virosa	0	Phyteuma nigrum	0
Erophila verna	1	Circaea alpina	0	Phyteuma orbiculare	0
Erysimum cheiranthoides	1	Circaea intermedia	0	Phyteuma ovatum	0
Erysimum repandum	1	Circaea lutetiana	0	Phyteuma spicatum	0
Eupatorium cannabinum	1	Cirsium acaule	0	Pimpinella major	0
Euphorbia cyparissias	1	Cirsium canum	0	Pimpinella nigra	0
Euphorbia epithymoides	1	Cirsium eriophorum	0	Pimpinella saxifraga	0
Euphorbia esula	1	Cirsium helenioides	0	Pinguicula alpina	0
Euphorbia platyphyllos	1	Cirsium rivulare	0	Pinguicula vulgaris	0

Filipendula ulmaria	1	Cirsium spinosissimum	0	Plantago alpina	0
Fragaria vesca	1	Cirsium tuberosum	0	Plantago atrata	0
Galanthus nivalis	1	Clematis recta	0	Plantago intermedia	0
Galeopsis tetrahit	1	Cochlearia pyrenaica	0	Plantago maritima	0
Galium aparine	1	Coeloglossum viride	0	Plantago media	0
Galium palustre	1	Colchicum autumnale	0	Plantago strictissima	0
Galium verum	1	Coleanthus subtilis	0	Plantago winteri	0
Genista tinctoria	1	Corallorrhiza trifida	0	Platanthera bifolia	0
Geranium lucidum	1	Cortusa matthioli	0	Platanthera chlorantha	0
Geranium robertianum	1	Corydalis cava	0	Pleurospermum austriacum	0
Geranium sanguineum	1	Corydalis intermedia	0	Poa alpina	0
Glechoma hederacea	1	Corydalis pumila	0	Poa angustifolia	0
Glyceria declinata	1	Corydalis solida	0	Poa badensis	0
Holcus lanatus	1	Crassula aquatica	0	Poa cenisia	0
Holcus mollis	1	Crepis alpestris	0	Poa chaixii	0
Holosteum umbellatum	1	Crepis aurea	0	Poa glauca	0
Humulus lupulus	1	Crepis bocconi	0	Poa humilis	0
Hypericum humifusum	1	Crepis conyzifolia	0	Poa hybrida	0
Hypericum perforatum	1	Crepis jacquinii	0	Poa minor	0
Hypericum tetrapterum	1	Crepis mollis	0	Poa nemoralis	0
Hypochaeris glabra	1	Crepis paludosa	0	Poa remota	0
Hypochaeris radicata	1	Crepis praemorsa	0	Poa supina	0
Illecebrum verticillatum	1	Crepis pyrenaica	0	Podospermum laciniatum	0
Impatiens noli-tangere	1	Crepis terglouensis	0	Polemonium caeruleum	0
Iris pseudacorus	1	Crocus albiflorus	0	Polycnemum verrucosum	0
Iris spuria	1	Cruciata glabra	0	Polygala alpestris	0
Juncus acutiflorus	1	Cucubalus baccifer	0	Polygala chamaebuxus	0
Juncus articulatus	1	Cuscuta europaea	0	Polygala comosa	0
Juncus bufonius	1	Cuscuta lupuliformis	0	Polygonatum multiflorum	0
Juncus capitatus	1	Cyclamen purpurascens	0	Polygonatum odoratum	0
Juncus compressus	1	Cynoglossum germanicum	0	Polygonatum verticillatum	0
Juncus effusus	1	Cyperus flavescens	0	Potentilla alba	0
Juncus gerardii	1	Cyperus fuscus	0	Potentilla anglica	0
Juncus inflexus	1	Cypripedium calceolus	0	Potentilla anserina	0
Juncus maritimus	1	Dactylis polygama	0	Potentilla aurea	0
Juncus squarrosus	1	Dactylorhiza cruenta	0	Potentilla brauneana	0
Knautia arvensis	1	Dactylorhiza fuchsii	0	Potentilla caulescens	0
Lactuca saligna	1	Dactylorhiza incarnata	0	Potentilla clusiana	0
Lactuca serriola	1	Dactylorhiza lapponica	0	Potentilla collina	0
Lactuca virosa	1	Dactylorhiza maculata	0	Potentilla crantzii	0
Lamium maculatum	1	Dactylorhiza majalis	0	Potentilla incana	0
Lapsana communis	1	Dactylorhiza sambucina	0	Potentilla micrantha	0
Lathyrus sylvestris	1	Dactylorhiza traunsteineri	0	Potentilla mixta	0
Leontodon autumnalis	1	Danthonia alpina	0	Potentilla neglecta	0
Leontodon hispidus	1	Daphne cneorum	0	Potentilla pusilla	0
Leontodon saxatilis	1	Daphne striata	0	Potentilla rupestris	0
Lepidium latifolium	1	Dentaria bulbifera	0	Potentilla sterilis	0
Leucanthemum vulgare	1	Dentaria enneaphyllos	0	Potentilla thuringiaca	0
Linaria vulgaris	1	Dentaria pentaphyllos	0	Potentilla thyrsiflora	0
Linum catharticum	1	Deschampsia cespitosa	0	Prenanthes purpurea	0
Lolium perenne	1	Deschampsia littoralis	0	Primula auricula	0
Lotus corniculatus	1	Dianthus carthusianorum	0	Primula clusiana	0
Lotus pedunculatus	1	Dianthus deltoides	0	Primula elatior	0
Ludwigia palustris	1	Dianthus seguieri	0	Primula farinosa	0
Lunaria rediviva	1	Dianthus superbus	0	Primula hirsuta	0
Luzula luzuloides	1	Dianthus sylvestris	0	Primula minima	0
Luzula sylvatica	1	Dichostylis micheliana	0	Primula veris	0
Lychnis flos-cuculi	1	Dictamnus albus	0	Primula vulgaris	0
Lysimachia nummularia	1	Digitalis grandiflora	0	Pseudolysimachion longifolium	0
Lysimachia thyrsiflora	1	Digitalis lutea	0	Pseudorchis albida	0
Lysimachia vulgaris	1	Doronicum austriacum	0	Psilathera ovata	0
Lythrum hyssopifolia	1	Doronicum columnae	0	Puccinellia distans	0

Lythrum salicaria	1	Doronicum glaciale	0	Puccinellia limosa	0
Medicago falcata	1	Doronicum grandiflorum	0	Pulicaria dysenterica	0
Medicago lupulina	1	Doronicum pardalianches	0	Pulicaria vulgaris	0
Medicago minima	1	Dorycnium germanicum	0	Pulmonaria angustifolia	0
Melica nutans	1	Dorycnium herbaceum	0	Pulmonaria mollis	0
Mentha aquatica	1	Drosera intermedia	0	Pulmonaria obscura	0
Mentha arvensis	1	Drosera longifolia	0	Pulmonaria officinalis	0
Mentha pulegium	1	Drosera obovata	0	Pulsatilla alpina	0
Mentha verticillata	1	Drosera rotundifolia	0	Pulsatilla micrantha	0
Mercurialis perennis	1	Dryas octopetala	0	Pulsatilla pratensis	0
Molinia caerulea	1	Elatine hexandra	0	Pulsatilla vernalis	0
Muscari botryoides	1	Elatine hydropiper	0	Pulsatilla vulgaris	0
Mycelis muralis	1	Eleocharis acicularis	0	Pyrola chlorantha	0
Myosotis discolor	1	Eleocharis austriaca	0	Pyrola media	0
Myosotis laxa	1	Eleocharis mamillata	0	Pyrola minor	0
Myosotis scorpioides	1	Eleocharis ovata	0	Pyrola rotundifolia	0
Myosotis sylvatica	1	Eleocharis quinqueflora	0	Radiola linoides	0
Myosoton aquaticum	1	Eleocharis uniglumis	0	Ranunculus aconitifolius	0
Narcissus pseudonarcissus	1	Elyna myosuroides	0	Ranunculus alpestris	0
Nardus stricta	1	Elytrigia atherica	0	Ranunculus argoviensis	0
Ononis repens	1	Empetrum hermaphroditum	0	Ranunculus breyninus	0
Ononis spinosa	1	Empetrum nigrum	0	Ranunculus carinthiacus	0
Origanum vulgare	1	Epilobium alpestre	0	Ranunculus cassubicifolius	0
Pastinaca sativa	1	Epilobium alsinifolium	0	Ranunculus dactylophyllus	0
Persicaria lapathifolia	1	Epilobium anagallidifolium	0	Ranunculus glacialis	0
Persicaria maculosa	1	Epilobium collinum	0	Ranunculus hybridus	0
Petrorhagia prolifera	1	Epilobium lamyi	0	Ranunculus illyricus	0
Phalaris arundinacea	1	Epilobium lanceolatum	0	Ranunculus lanuginosus	0
Phleum pratense	1	Epilobium montanum	0	Ranunculus megacarpus	0
Physalis alkekengi	1	Epilobium nutans	0	Ranunculus montanus	0
Picris hieracioides	1	Epilobium obscurum	0	Ranunculus nemorosus	0
Plantago major	1	Epilobium palustre	0	Ranunculus parnassiifolius	0
Poa annua	1	Epilobium parviflorum	0	Ranunculus phragmiteti	0
Poa bulbosa	1	Epilobium roseum	0	Ranunculus platanifolius	0
Poa compressa	1	Epilobium tetragonum	0	Ranunculus polyanthemoides	0
Poa palustris	1	Epipactis albensis	0	Ranunculus polyanthemophyllus	0
Poa pratensis	1	Epipactis atrorubens	0	Ranunculus polyanthemos	0
Poa trivialis	1	Epipactis greuteri	0	Ranunculus reptans	0
Polygonum arenastrum	1	Epipactis leptochila	0	Ranunculus serpens	0
Polygonum aviculare	1	Epipactis microphylla	0	Ranunculus villarsii	0
Potentilla argentea	1	Epipactis muelleri	0	Rapistrum perenne	0
Potentilla erecta	1	Epipactis palustris	0	Rhamnus pumila	0
Potentilla reptans	1	Epipogium aphyllum	0	Rhinanthus alectorolophus	0
Potentilla supina	1	Erica carnea	0	Rhinanthus angustifolius	0
Pseudognaphalium luteoalbum	1	Erigeron acris	0	Rhinanthus glacialis	0
Ranunculus acris	1	Erigeron alpinus	0	Rhinanthus minor	0
Ranunculus bulbosus	1	Erigeron atticus	0	Rhinanthus pulcher	0
Ranunculus ficaria	1	Erigeron glabratus	0	Rhodiola rosea	0
Ranunculus flammula	1	Erigeron neglectus	0	Rhodothamnus chamaecistus	0
Ranunculus lingua	1	Erigeron uniflorus	0	Rhynchospora alba	0
Ranunculus repens	1	Eriophorum angustifolium	0	Rhynchospora fusca	0
Ranunculus sardous	1	Eriophorum gracile	0	Roegneria canina	0
Ranunculus sceleratus	1	Eriophorum latifolium	0	Rorippa pyrenaica	0
Rorippa amphibia	1	Eriophorum scheuchzeri	0	Rumex alpinus	0
Rorippa austriaca	1	Eriophorum vaginatum	0	Rumex aquaticus	0
Rorippa palustris	1	Erucastrum nasturtiifolium	0	Rumex arifolius	0
Rorippa sylvestris	1	Eryngium campestre	0	Rumex hydrolapathum	0
Rumex acetosa	1	Eryngium planum	0	Rumex maritimus	0
Rumex acetosella	1	Erysimum marschallianum	0	Rumex palustris	0
Rumex conglomeratus	1	Erysimum odoratum	0	Rumex thyrsiflorus	0
Rumex crispus	1	Erysimum virgatum	0	Sagina nodosa	0
Rumex obtusifolius	1	Euphorbia dulcis	0	Sagina normaniana	0

Rumex sanguineus	1	Euphorbia lucida	0	Sagina saginoides	0
Salsola kali	1	Euphorbia palustris	0	Salicornia europaea	0
Salvia nemorosa	1	Euphorbia salicifolia	0	Salix alpina	0
Salvia pratensis	1	Euphorbia seguieriana	0	Salix breviserrata	0
Sambucus ebulus	1	Euphorbia stricta	0	Salix herbacea	0
Samolus valerandi	1	Euphorbia verrucosa	0	Salix myrtilloides	0
Sanguisorba minor	1	Euphorbia villosa	0	Salix reticulata	0
Saponaria ocymoides	1	Euphrasia hirtella	0	Salix retusa	0
Saponaria officinalis	1	Euphrasia micrantha	0	Salix serpillifolia	0
Sclerochloa dura	1	Euphrasia minima	0	Salvia glutinosa	0
Securigera varia	1	Euphrasia nemorosa	0	Sanguisorba officinalis	0
Sedum telephium	1	Euphrasia officinalis	0	Sanicula europaea	0
Senecio jacobaea	1	Euphrasia salisburgensis	0	Saussurea alpina	0
Senecio sylvaticus	1	Euphrasia stricta	0	Saussurea discolor	0
Senecio vulgaris	1	Euphrasia tricuspidata	0	Saussurea pygmaea	0
Silene conica	1	Falcaria vulgaris	0	Saxifraga granulata	0
Silene latifolia	1	Fallopia dumetorum	0	Saxifraga rotundifolia	0
Solanum dulcamara	1	Filago arvensis	0	Saxifraga tridactylites	0
Sonchus arvensis	1	Filago lutescens	0	Scabiosa canescens	0
Sonchus asper	1	Filago minima	0	Scabiosa columbaria	0
Sonchus oleraceus	1	Filago vulgaris	0	Scabiosa lucida	0
Sonchus palustris	1	Filipendula vulgaris	0	Scabiosa ochroleuca	0
Spergula morisonii	1	Fragaria moschata	0	Scheuchzeria palustris	0
Spergula pentandra	1	Fragaria viridis	0	Schoenoplectus mucronatus	0
Spergularia media	1	Fumana procumbens	0	Schoenoplectus pungens	0
Stachys palustris	1	Gagea bohemica	0	Schoenoplectus supinus	0
Stellaria alsine	1	Gagea fragifera	0	Schoenoplectus triqueter	0
Stellaria graminea	1	Gagea lutea	0	Schoenus ferrugineus	0
Suaeda maritima	1	Gagea minima	0	Schoenus nigricans	0
Symphytum officinale	1	Gagea pomeranica	0	Scilla bifolia	0
Tamus communis	1	Gagea pratensis	0	Scirpoides holoschoenus	0
Teesdalia nudicaulis	1	Gagea spathacea	0	Scirpus radicans	0
Thlaspi perfoliatum	1	Galeopsis bifida	0	Scirpus sylvaticus	0
Thymelaea passerina	1	Galeopsis ladanum	0	Scleranthus perennis	0
Thymus pulegioides	1	Galeopsis pubescens	0	Scleranthus polycarpos	0
Thymus serpyllum	1	Galeopsis speciosa	0	Scleranthus verticillatus	0
Torilis japonica	1	Galium album	0	Scorzonera austriaca	0
Tragopogon dubius	1	Galium aristatum	0	Scorzonera hispanica	0
Tragopogon pratensis	1	Galium boreale	0	Scorzonera humilis	0
Trifolium arvense	1	Galium glaucum	0	Scorzonera parviflora	0
Trifolium aureum	1	Galium lucidum	0	Scorzonera purpurea	0
Trifolium campestre	1	Galium megalospermum	0	Scrophularia canina	0
Trifolium dubium	1	Galium pomeranicum	0	Scrophularia nodosa	0
Trifolium fragiferum	1	Galium pumilum	0	Scrophularia umbrosa	0
Trifolium hybridum	1	Galium schultesii	0	Scutellaria galericulata	0
Trifolium pratense	1	Galium sterneri	0	Scutellaria hastifolia	0
Trifolium repens	1	Galium sylvaticum	0	Scutellaria minor	0
Triglochin palustre	1	Galium truniacum	0	Sedum annuum	0
Tussilago farfara	1	Galium uliginosum	0	Sedum atratum	0
Valeriana officinalis	1	Galium valdepilosum	0	Sedum maximum	0
Ventenata dubia	1	Galium wirtgenii	0	Sedum villosum	0
Verbascum blattaria	1	Genista germanica	0	Selinum carvifolia	0
Verbascum thapsus	1	Genista pilosa	0	Senecio abrotanifolius	0
Veronica anagallis-aquatica	1	Genista sagittalis	0	Senecio alpinus	0
Veronica catenata	1	Gentiana acaulis	0	Senecio aquaticus	0
Veronica hederifolia	1	Gentiana asclepiadea	0	Senecio doronicum	0
Veronica serpyllifolia	1	Gentiana bavarica	0	Senecio erraticus	0
Veronica verna	1	Gentiana clusii	0	Senecio erucifolius	0
Vicia cracca	1	Gentiana cruciata	0	Senecio germanicus	0
Vicia hirsuta	1	Gentiana lutea	0	Senecio hercynicus	0
Vicia sepium	1	Gentiana nivalis	0	Senecio incanus	0
Vicia tetrasperma	1	Gentiana orbicularis	0	Senecio ovatus	0

Viola riviniana	1	Gentiana pannonica	0	Senecio paludosus	0
Vulpia bromoides	1	Gentiana pneumonanthe	0	Senecio rupestris	0
Aceras anthropophorum	0	Gentiana punctata	0	Senecio sarracenicus	0
Achillea atrata	0	Gentiana purpurea	0	Senecio subalpinus	0
Achillea clavennae	0	Gentiana utriculosa	0	Senecio viscosus	0
Achillea collina	0	Gentiana verna	0	Serratula tinctoria	0
Achillea macrophylla	0	Gentianella amarella	0	Seseli annuum	0
Achillea pannonica	0	Gentianella aspera	0	Seseli hippomarathrum	0
Achillea pratensis	0	Gentianella bohemica	0	Seseli libanotis	0
Achillea salicifolia	0	Gentianella campestris	0	Sesleria albicans	0
Achillea setacea	0	Gentianella ciliata	0	Sibbaldia procumbens	0
Acinos alpinus	0	Gentianella germanica	0	Silaum silaus	0
Aconitum austriacum	0	Gentianella lutescens	0	Silene armeria	0
Aconitum degenii	0	Gentianella tenella	0	Silene dioica	0
Aconitum hebegynum	0	Geranium bohemicum	0	Silene nutans	0
Aconitum lycoctonum	0	Geranium palustre	0	Silene otites	0
Aconitum pilipes	0	Geranium phaeum	0	Silene rupestris	0
Aconitum pilosiusculum	0	Geranium pratense	0	Silene viscosa	0
Aconitum tauricum	0	Geranium sylvaticum	0	Sisymbrium austriacum	0
Aconitum variegatum	0	Geum montanum	0	Sisymbrium strictissimum	0
Actaea spicata	0	Geum reptans	0	Sium latifolium	0
Adenophora liliifolia	0	Geum rivale	0	Soldanella alpicola	0
Adenostyles alliariae	0	Geum urbanum	0	Soldanella alpina	0
Adenostyles glabra	0	Gladiolus imbricatus	0	Soldanella austriaca	0
Adonis vernalis	0	Gladiolus palustris	0	Soldanella minima	0
Agrimonia eupatoria	0	Glaux maritima	0	Soldanella montana	0
Agrimonia procera	0	Globularia cordifolia	0	Solidago virgaurea	0
Agrostis agrostiflora	0	Globularia nudicaulis	0	Spergularia echinosperma	0
Agrostis alpina	0	Gnaphalium hoppeanum	0	Spergularia salina	0
Agrostis rupestris	0	Gnaphalium norvegicum	0	Spiranthes aestivalis	0
Agrostis schleicheri	0	Gnaphalium supinum	0	Spiranthes spiralis	0
Agrostis vinealis	0	Gnaphalium sylvaticum	0	Stachys alpina	0
Ajuga pyramidalis	0	Gnaphalium uliginosum	0	Stachys germanica	0
Allium angulosum	0	Goodyera repens	0	Stachys recta	0
Allium cirrhosum	0	Gratiola officinalis	0	Stachys sylvatica	0
Allium kochii	0	Gymnadenia conopsea	0	Stellaria longifolia	0
Allium lusitanicum	0	Gymnadenia odoratissima	0	Stellaria neglecta	0
Allium rotundum	0	Gypsophila muralis	0	Stellaria nemorum	0
Allium schoenoprasum	0	Hammarbya paludosa	0	Stellaria palustris	0
Allium sphaerocephalon	0	Hedysarum hedysaroides	0	Stipa borysthenica	0
Allium strictum	0	Helianthemum alpestre	0	Stipa capillata	0
Allium suaveolens	0	Helianthemum apenninum	0	Stipa dasyphylla	0
Allium ursinum	0	Helianthemum canum	0	Stipa eriocaulis	0
Allium victorialis	0	Helianthemum nummularium	0	Stipa pennata	0
Alopecurus rendlei	0	Helichrysum arenarium	0	Stipa pulcherrima	0
Althaea officinalis	0	Helictotrichon parlatorei	0	Stipa tirsa	0
Anacamptis pyramidalis	0	Helictotrichon pratense	0	Streptopus amplexifolius	0
Anagallis foemina	0	Helictotrichon pubescens	0	Succisa pratensis	0
Andromeda polifolia	0	Helictotrichon versicolor	0	Swertia perennis	0
Androsace lactea	0	Helleborus niger	0	Symphytum tuberosum	0
Androsace septentrionalis	0	Helleborus viridis	0	Tanacetum corymbosum	0
Anemone narcissiflora	0	Hepatica nobilis	0	Tephroseris crispa	0
Anemone nemorosa	0	Heracleum austriacum	0	Tephroseris helenitis	0
Anemone ranunculoides	0	Heracleum sphondylium	0	Tephroseris integrifolia	0
Anemone sylvestris	0	Herminium monorchis	0	Tephroseris tenuifolia	0
Angelica archangelica	0	Hierochloe australis	0	Tetragonolobus maritimus	0
Angelica palustris	0	Hierochloe hirta	0	Teucrium chamaedrys	0
Antennaria carpatica	0	Hierochloe odorata	0	Teucrium montanum	0
Anthericum liliago	0	Himantoglossum hircinum	0	Teucrium scordium	0
Anthericum ramosum	0	Hippocrepis comosa	0	Thalictrum aquilegiifolium	0
Anthoxanthum alpinum	0	Homogyne alpina	0	Thalictrum flavum	0
Anthriscus nitida	0	Homogyne discolor	0	Thalictrum lucidum	0

Anthyllis vulneraria	0	Hordelymus europaeus	0	Thalictrum minus	0
Apera spica-venti	0	Horminum pyrenaicum	0	Thalictrum simplex	0
Apium repens	0	Hornungia petraea	0	Thesium alpinum	0
Aposeris foetida	0	Hyacinthoides non-scripta	0	Thesium bavarum	0
Aquilegia atrata	0	Hydrocotyle vulgaris	0	Thesium ebracteatum	0
Aquilegia einseleana	0	Hypericum elegans	0	Thesium linophyllon	0
Arabis auriculata	0	Hypericum hirsutum	0	Thesium pyrenaicum	0
Arabis caerulea	0	Hypericum maculatum	0	Thesium rostratum	0
Arabis glabra	0	Hypericum montanum	0	Thymus praecox	0
Arabis hirsuta	0	Hypericum pulchrum	0	Tofieldia calyculata	0
Arabis nemorensis	0	Hypochaeris maculata	0	Tofieldia pusilla	0
Arabis pauciflora	0	Hypochaeris uniflora	0	Tolpis staticifolia	0
Arabis sagittata	0	Inula britannica	0	Tozzia alpina	0
Arabis turrita	0	Inula conyzae	0	Tragopogon orientalis	0
Arctium nemorosum	0	Inula germanica	0	Traunsteinera globosa	0
Arctostaphylos alpinus	0	Inula hirta	0	Trichophorum alpinum	0
Arctostaphylos uva-ursi	0	Inula salicina	0	Trichophorum cespitosum	0
Aremonia agrimonoides	0	Iris sibirica	0	Trientalis europaea	0
Arenaria biflora	0	Iris variegata	0	Trifolium alpestre	0
Arenaria ciliata	0	Isolepis setacea	0	Trifolium badium	0
Arnica montana	0	Jasione montana	0	Trifolium medium	0
Arnoseris minima	0	Juncus alpinus	0	Trifolium montanum	0
Artemisia laciniata	0	Juncus atratus	0	Trifolium ochroleucon	0
Artemisia scoparia	0	Juncus conglomeratus	0	Trifolium retusum	0
Artemisia umbelliformis	0	Juncus filiformis	0	Trifolium rubens	0
Arum maculatum	0	Juncus jacquinii	0	Trifolium spadiceum	0
Asarum europaeum	0	Juncus minutulus	0	Trifolium thalii	0
Asperula cynanchica	0	Juncus ranarius	0	Triglochin maritimum	0
Asperula tinctoria	0	Juncus sphaerocarpus	0	Trinia glauca	0
Aster alpinus	0	Juncus subnodulosus	0	Trisetum distichophyllum	0
Aster amellus	0	Juncus tenageia	0	Trisetum flavescens	0
Aster bellidiastrum	0	Juncus trifidus	0	Trisetum spicatum	0
Aster linosyris	0	Juncus triglumis	0	Trollius europaeus	0
Aster tripolium	0	Juniperus sibirica	0	Urtica kioviensis	0
Astragalus alpinus	0	Knautia dipsacifolia	0	Vaccinium myrtillus	0
Astragalus australis	0	Knautia drymeia	0	Vaccinium oxycoccos	0
Astragalus cicer	0	Knautia kitaibelii	0	Vaccinium uliginosum	0
Astragalus danicus	0	Kobresia simpliciuscula	0	Vaccinium vitis-idaea	0
Astragalus exscapus	0	Koeleria glauca	0	Valeriana dioica	0
Astragalus frigidus	0	Koeleria macrantha	0	Valeriana montana	0
Astragalus glycyphyllos	0	Koeleria pyramidata	0	Valeriana pratensis	0
Astragalus penduliflorus	0	Lactuca perennis	0	Valeriana procurrens	0
Astrantia bavarica	0	Lactuca quercina	0	Valeriana sambucifolia	0
Astrantia major	0	Lactuca viminea	0	Valeriana saxatilis	0
Athamanta cretensis	0	Lappula deflexa	0	Valeriana supina	0
Atriplex littoralis	0	Laser trilobum	0	Valeriana versifolia	0
Atropa bella-donna	0	Laserpitium latifolium	0	Valeriana wallrothii	0
Barbarea stricta	0	Laserpitium prutenicum	0	Valerianella carinata	0
Bartsia alpina	0	Laserpitium siler	0	Valerianella dentata	0
Bassia laniflora	0	Lathraea squamaria	0	Valerianella rimosa	0
Betonica alopecuros	0	Lathyrus heterophyllus	0	Veratrum album	0
Betonica officinalis	0	Lathyrus laevigatus	0	Verbascum densiflorum	0
Biscutella laevigata	0	Lathyrus linifolius	0	Verbascum lychnitis	0
Bistorta officinalis	0	Lathyrus niger	0	Verbascum nigrum	0
Bistorta vivipara	0	Lathyrus palustris	0	Verbascum phlomoides	0
Blackstonia acuminata	0	Lathyrus pannonicus	0	Verbascum phoeniceum	0
Blackstonia perfoliata	0	Lathyrus pratensis	0	Verbascum pulverulentum	0
Blysmus compressus	0	Lathyrus tuberosus	0	Veronica acinifolia	0
Bolboschoenus maritimus	0	Lathyrus vernus	0	Veronica alpina	0
Bolboschoenus maritimus	0	Lavatera thuringiaca	0	Veronica anagalloides	0
Bolboschoenus yagara	0	Leontodon helveticus	0	Veronica aphylla	0
Brachypodium rupestre	0	Leontodon incanus	0	Veronica bellidioides	0

Briza media	0	Leontodon montanus	0	Veronica dillenii	0
Bromus benekenii	0	Leontopodium alpinum	0	Veronica fruticans	0
Bromus lepidus	0	Leonurus marrubiastrum	0	Veronica fruticulosa	0
Bromus ramosus	0	Lepidium graminifolium	0	Veronica montana	0
Buglossoides purpurocaerulea	0	Leucanthemopsis alpina	0	Veronica praecox	0
Buphthalmum salicifolium	0	Leucanthemum adustum	0	Veronica scutellata	0
Bupleurum falcatum	0	Leucanthemum halleri	0	Veronica teucrium	0
Bupleurum longifolium	0	Leucanthemum ircutianum	0	Vicia cassubica	0
Bupleurum ranunculoides	0	Leucojum vernum	0	Vicia dumetorum	0
Bupleurum tenuissimum	0	Ligusticum mutellina	0	Vicia lathyroides	0
Calamagrostis arundinacea	0	Ligusticum mutellinoides	0	Vicia oroboides	0
Calamagrostis canescens	0	Lilium bulbiferum	0	Vicia pisiformis	0
Calamagrostis pseudophragmites	0	Lilium martagon	0	Vicia sylvatica	0
Calamagrostis varia	0	Limodorum abortivum	0	Vicia tenuifolia	0
Calamagrostis villosa	0	Limosella aquatica	0	Vincetoxicum hirundinaria	0
Calamintha einseleana	0	Linaria alpina	0	Viola alba	0
Calamintha menthifolia	0	Lindernia procumbens	0	Viola ambigua	0
Calamintha nepeta	0	Linum alpinum	0	Viola biflora	0
Calla palustris	0	Linum flavum	0	Viola calcarata	0
Caltha palustris	0	Linum perenne	0	Viola canina	0
Campanula alpina	0	Linum tenuifolium	0	Viola collina	0
Campanula barbata	0	Linum viscosum	0	Viola elatior	0
Campanula baumgartenii	0	Liparis loeselii	0	Viola hirta	0
Campanula bononiensis	0	Listera cordata	0	Viola kitaibeliana	0
Campanula cervicaria	0	Listera ovata	0	Viola mirabilis	0
Campanula cochleariifolia	0	Lithospermum officinale	0	Viola montana	0
Campanula glomerata	0	Lloydia serotina	0	Viola palustris	0
Campanula latifolia	0	Loiseleuria procumbens	0	Viola pumila	0
Campanula patula	0	Lomatogonium carinthiacum	0	Viola pyrenaica	0
Campanula persicifolia	0	Lotus tenuis	0	Viola reichenbachiana	0
Campanula rapunculus	0	Luzula alpina	0	Viola rupestris	0
Campanula rotundifolia	0	Luzula alpinopilosa	0	Viola schultzii	0
Campanula scheuchzeri	0	Luzula campestris	0	Viola stagnina	0
Campanula sibirica	0	Luzula divulgata	0	Viola tricolor	0
Campanula thyrsoides	0	Luzula forsteri	0	Virga pilosa	0
Campanula trachelium	0	Luzula glabrata	0		
Cardamine alpina	0	Luzula luzulina	0		
Cardamine amara	0	Luzula multiflora	0		
Cardamine dentata	0	Luzula nivea	0		
Cardamine parviflora	0	Luzula pallidula	0		
Cardamine resedifolia	0	Luzula pilosa	0		
Cardamine trifolia	0	Luzula spicata	0		
Cardamine udicola	0	Luzula sudetica	0		
